# Changes in epidemiological characteristics of knee arthroplasty in eastern, northern and central China between 2011 and 2020

**DOI:** 10.1186/s13018-023-03600-3

**Published:** 2023-02-14

**Authors:** Weiyi Sun, Peizhi Yuwen, Xuemei Yang, Wei Chen, Yingze Zhang

**Affiliations:** 1 Department of Emergency, Third Hospital of Hebei Medical University, Shijiazhuang, 050051 Hebei People’s Republic of China; 2 Key Laboratory of Biomechanics of Hebei Province, Shijiazhuang, 050051 People’s Republic of China; 3 Department of Obstetrics, Shijiazhuang Obstetrics and Gynecology Hospital, The Fourth Hospital of Shijiazhuang, Shijiazhuang, 050051 People’s Republic of China; 4 Department of Orthopedic Surgery, The Third Hospital of Hebei Medical University, No. 139 Ziqiang Road, Qiaoxi District, Shijiazhuang, 050051 Hebei Province People’s Republic of China; 5 NHC Key Laboratory of Intelligent Orthopaedic Equipment, Shijiazhuang, 050051 People’s Republic of China; 6 Chinese Academy of Engineering, Beijing, 100088 People’s Republic of China

**Keywords:** Knee osteoarthritis, Knee arthroplasty, Epidemiology, Changes, Characteristics

## Abstract

**Objective:**

To explore changes in the epidemiological and clinical characteristics of patients who underwent knee arthroplasty (KA) over a 10-year period in China.

**Methods:**

Medical records of patients with knee osteoarthritis (KOA), who underwent primary unilateral KA in 5 level I center hospitals in China between January 2011 and December 2020, were retrospectively reviewed and analyzed. To more clearly define changes over the years, patients were divided into two groups according to time of admission at 5-year intervals. Age, sex, body mass index (BMI), Kellgren–Lawrence (K–L) classification, comorbid diseases, surgical procedures, hospital stay, and hospitalization costs were compared between the two groups.

**Results:**

A total of 23,610 patients with KOA (5400 male and 18,210 females; mean age: 65.7 ± 7.6 years) who underwent primary unilateral KA were included. The number of KAs increased in recent years (group A, *n* = 7606 vs. group B, *n* = 16,004). Significant differences were noted in age, sex, BMI, K–L classification, comorbidities, surgical procedures, hospital stay, and hospitalization costs between the two periods (*P* < 0.05). More than three-quarters of KA cases involved females, and the age at surgery tended to be younger than that reported in foreign countries. In group B, the proportion of overweight and grade III, number of comorbidities, and unicompartmental knee arthroplasty patients increased compared to that in group A; however, hospitalization costs and length of hospital stay decreased.

**Conclusions:**

Results suggested that the epidemiological characteristics of patients undergoing KA have changed over time. An analysis of the epidemiological characteristics of patients undergoing KA treatment may provide a scientific basis for the prevention and control of KOA.

## Introduction

Knee osteoarthritis (KOA) is a chronic and degenerative joint disease that occurs mostly in middle-aged and elderly individuals [1]. Knee pain is the main clinical manifestation and can be accompanied by difficulties with ambulation. In severe cases, the ability to walk can be lost, seriously affecting the quality of life of those affected, thus creating a huge and lasting burden on individuals and society [2]. The number of patients with KOA is increasing with the aging population of China. The latest survey results revealed that the prevalence of KOA in China is 8.1% [3].

Knee arthroplasty (KA), including total knee arthroplasty (TKA) and unicompartmental knee arthroplasty (UKA), is considered to be the best treatment for patients with end-stage KOA, with high safety and efficacy profiles [4]. When deciding whether to undergo KA, patients and surgeons need to choose which type of replacement procedure to use: TKA or UKA. Generally, the key indications for KA are considered to be moderate to severe knee arthritis (grade III–IV according the Kellgren–Lawrence (K–L) classification) and persistent severe pain. Indications for UKA are the same as for TKA, but the disease should also be isolated in one compartment, ligaments need to be intact, and any deformity should be correctable [5]. Replacing the diseased articular surface with an artificial prosthesis can relieve pain and improves knee function [6]. A total of 650,000 patients underwent TKA in the USA in 2008, 77,500 in the United Kingdom in 2009, and 103,601 in South Korea between 2002 and 2005 [7]. According to current research, there have been many reports addressing the epidemiology of KA in Europe, the USA, and other countries. In recent years, the field of KA has rapidly developed in China. However, no similar reports have described the epidemiology of patients undergoing KA treatment in China. Therefore, a large-scale multicenter study is needed to understand the epidemiological characteristics of patients undergoing KA for knee arthritis in China.

To comprehensively understand the epidemiological characteristics of KA in China, information from patients who underwent primary unilateral KA due to KOA in five Level I center hospitals in China over the past 10 years was retrospectively collected. Trends in age, sex, body mass index (BMI), K–L classification, comorbidities, surgical procedures, length of hospital stay, and hospitalization costs were analyzed.

## Materials and methods

The present study was conducted in accordance to the principles of the Declaration of Helsinki. The study protocol was approved by the ethics committee of the five participating hospitals. A retrospective analysis of 23,610 patients with KOA, who underwent KA at five Level I center hospitals in east, north, and central China between January 2011 and December 2020, was performed. Inclusion criteria were as follows: patients who underwent primary unilateral KA (TKA or UKA) due to KOA; and patients who provided informed written consent for the treatment plan and agreed to undergo postoperative follow-up. Individuals with rheumatoid arthritis or other inflammatory diseases; those who underwent previous KA, patients with physical, neurological, or mental conditions that could compromise compliance with postoperative rehabilitation and follow-up, and those with known sensitivity to the internal implant material used in the surgery were excluded.

### Data collection

To evaluate clinical changes more clearly, patients were divided into two groups according to the time of admission at 5-year intervals: group A (2011–2015); and group B (2016–2020). After reviewing the medical records of all patients with KOA, clinical data were analyzed according to the number of inpatients admitted. Data extracted included age, sex, BMI, K–L classification, combined diseases, surgical procedures, hospital stay, and hospitalization costs. To further investigate age at high incidence, patients were divided into age groups spanning 10 years. BMI categories were defined as follows: underweight, ≤ 18.5 kg/m^2^; normal, 18.5 to 23.9 kg/m^2^; overweight, 24.0 to 27.9 kg/m^2^; and obese, ≥ 28 kg/m^2^.

### Statistical analysis

All statistical analyses were performed using the SPSS version 26.0 (IBM Corporation., Armonk, NY, USA). Continuous variables are expressed as mean and standard deviation (SD) and were compared using the independent samples Student’s *t* test or Mann–Whitney *U* test, depending on whether the variables were normally distributed. Categorical variables are expressed as number and percentage (%) and were compared using the chi-squared test or Fisher’s exact test, as appropriate. Data that did not conform to a normal distribution expressed as median and interquartile range, and comparisons between groups were performed using the rank-sum test. Differences with *P* < 0.05 were considered to be statistically significant.

## Results

### Demographic characteristics and baseline data of enrolled patients

**Fig. 1:**
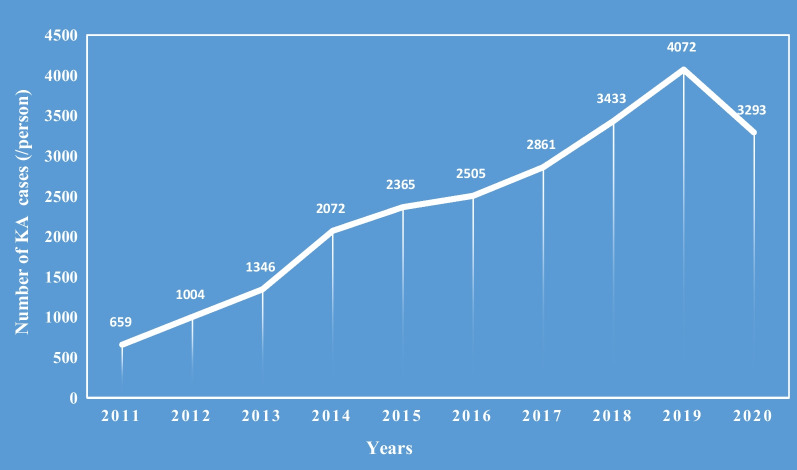
Annual cases (/person) undergoing KA in 5 level I center hospitals at Northern, Eastern and Central China during 2011–2020 (*n* = 23,610)

Overall, 23,610 patients with KOA who underwent KA in the five hospitals over the past 10 years were enrolled. The number of patients who underwent KA increased annually, except for a decrease in 2019–2020 due to the impact of the coronavirus diseases 2019 (COVID-19) pandemic (Fig. 1). There were 5400 male and 18,210 females, with a male-to-female ratio of 1:3.37; the mean age was 65.7 years (range 20–93 years). Among these, patients 60–69 years of age accounted for the highest proportion (46.5%, 10,980/23,610), followed by those 70–79 years, accounting for 26.6% (6274/23,610). Regarding surgical strategies, 22,288 (94.4%) patients underwent TKA and 1321 (5.6%) underwent UKA. The mean BMI of the patients was 26.4 ± 3.8 kg/m^2^ (range 14.6–57.8 kg/m^2^). Overweight individuals accounted for the highest proportions (42.1%), followed by obese patients (32.3%). According to the K–L classification, 22,128 (93.7%) patients were grade IV and 1482 (6.3%) patients were grade III. A total of 16,403 (69.5%) patients had a medical disease. Common comorbidities included hypertension, diabetes, coronary heart disease, and cerebrovascular disease. Classified according to the number of comorbidities, 8185 (34.7%) cases were combined with one medical disease, 4586 (19.4%) were combined with two medical diseases, and 3632 (15.4%) were combined with ≥ 3 medical diseases. The length of hospital stay of patients who underwent TKA ranged from 1 to 104 days, with a median of 11 days and an interquartile range of 8 days. Hospitalization costs ranged from $1336.20 to $40,048.10, with a median of $8393.90 and an interquartile range of $2460.00. The length of hospital stay of patients who underwent UKA ranged from 4 to 38 days, with a median of 9 days and an interquartile range of 6 days. Hospitalization costs ranged from $1525.20 to $20,122.80, with a median of $7319.40 and an interquartile range of $2662.60 (Table 1).

**Table 1:** Baseline data of patients with knee arthroplasty from 2011 to 2020

Variables	Number of patients (23,610)
Sex	
Male, *n* (%)	5400 (22.9)
Female, *n* (%)	18,210 (77.1)
Age (years, mean ± SD)	65.7 ± 7.6
≤ 19, *n* (%)	2 (0.0)
20–29, *n* (%)	16 (0.1)
30–39, *n* (%)	32 (0.1)
40–49, *n* (%)	563 (2.4)
50–59, *n* (%)	5060 (21.4)
70–79, *n* (%)	6274 (26.6)
80–89, *n* (%)	676 (2.9)
≥ 90, *n* (%)	7 (0.0)
BMI (kg/m^2^, mean ± SD)	26.4 ± 3.8
≤ 18.5, *n* (%)	245 (1.0)
18.5–23.9, *n* (%)	5795 (24.6)
24–27.9, *n* (%)	9936 (42.1)
≥ 28, *n* (%)	7634 (32.3)
Kellgren–Lawrence classification	
Grade III	1482 (6.3)
Grade IV	22,128 (93.7)
Surgical side	
Left, *n* (%)	11,269 (47.7)
Right, *n* (%)	12,341 (52.3)
Surgical strategies	
TKA, *n* (%)	22,288 (94.4)
UKA, *n* (%)	1321 (5.6)
Comorbidities	
0, *n* (%)	7207 (30.5)
1, *n* (%)	8185 (34.7)
2, *n* (%)	4586 (19.4)
≥ 3, *n* (%)	3632 (15.4)
Hospital stay (days)	
TKA (median, interquartile range)	11 (8)
UKA (median, interquartile range)	9 (6)
Hospitalization costs ($)	
TKA (median, interquartile range)	8393.9 (2,460.0)
UKA (median, interquartile range)	7319.4 (2,662.6)

*SD* standard deviation, *BMI* body mass index, *TKA* total knee arthroplasty, *UKA* unicompartmental knee arthroplasty

### Comparison of age and sex between the two periods

Between 2011 and 2015, 7606 KA patients were enrolled, including 1641 males and 5965 females. The male-to-female ratio in this group was 1:3.63, with a mean age of 65.4 years (range 23–93 years). Between 2016 and 2020, 16,004 patients with KA were included, including 3759 males and 12,245 females. The male-to-female ratio in this group was 1:3.26, with a mean age of 65.9 years (range 20–92 years). Although the age of patients in group B did not increase significantly compared with group A, there was statistically significant difference (group A, 65.4 ± 7.9 years vs. group B, 65.9 ± 7.4 years; *P* < 0.001). A significant difference was noted in sex between the two groups, with the number of male patients in group B significantly higher than that in group A (*P* = 0.001).

**Fig. 2:**
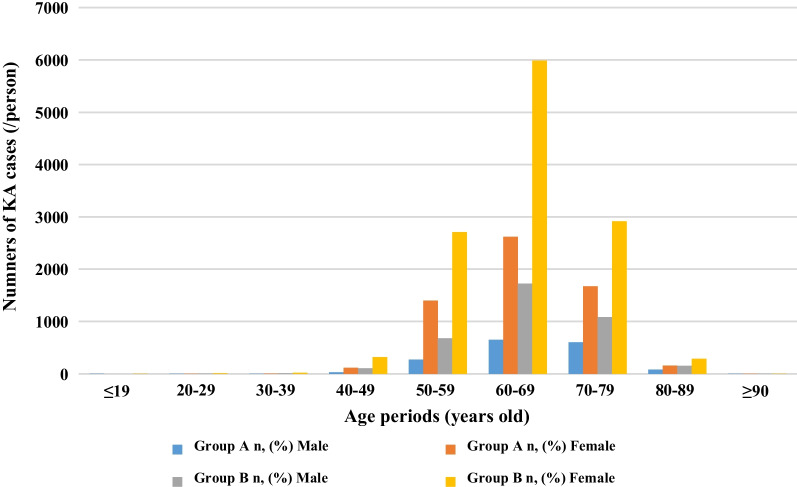
The age and sex distribution of patients undergoing KA in 5 level I center hospitals at Northern, Eastern and Central China during 2011–2020 (*n* = 23,610)

In group A, the proportion of those 60–69 years of age was highest. Among male patients, the proportion in this age group was 39.6% (650/1641); among female patients, the proportion was 43.9% (2618/5965). In group B, the 60–69 years’ age group accounted for the largest number of patients. Among male patients, the proportion in this age group increased from 39.6% to 45.9% (1725/3759). Among female patients, the proportion in this age group increased to 48.9% (5987/12,245). In addition, in the two periods, patients 50–59 years and 60–69 years of age, the proportion of female patients was significantly higher than that of male patients (Fig. 2). A significant difference was found in the male-to-female ratio between these two age groups (*P* = 0.001 and *P* = 0.004, respectively). However, the difference in the male-to-female ratio in other age groups was not statistically significant (Table 2).

**Table 2:** Comparison of gender composition ratio at all ages in Group A and Group B patients undergoing knee arthroplasty, n (%)

Age period (years old)	Group A *n*, (%)		Group B *n*, (%)		*P* value
	Male	Female	Male	Female	
≤ 19	1 (0.1)	0 (0.0)	0 (0.0)	1 (0.0)	0.157
20–29	1 (0.1)	1 (0.0)	6 (0.2)	8 (0.1)	0.849
30–39	2 (0.1)	4 (0.1)	9 (0.2)	17 (0.1)	0.952
40–49	28 (1.7)	112 (1.9)	105 (2.8)	318 (2.6)	0.244
50–59	270 (16.4)	1400 (23.5)	678 (18.0)	2712 (22.2)	0.001
60–69	650 (39.6)	2618 (43.9)	1725 (45.9)	5987 (48.9)	0.004
70–79	604 (36.8)	1674 (28.0)	1082 (28.8)	2914 (23.8)	0.629
80–89	84 (5.1)	155 (2.6)	151 (4.0)	286 (2.3)	0.877
≥ 90	1 (0.1)	1 (0.0)	3 (0.1)	2 (0.0)	0.809
Total	1641 (100.0)	5965 (100.0)	3759 (100.0)	12245 (100.0)	0.001

### Comparison of changes in BMI between the two periods

Regarding BMI of the enrolled patients, the number of overweight and obese patients in group A was the highest, accounting for 70.9% (5393/7606), followed by normal-weight patients accounting for 27.9% (2122/7606). In group B, overweight and obesity accounted for 76.0% (12,177/16,004), followed by normal-weight patients, accounting for 23.0% (3673/16,004). The proportion of overweight and obese patients in group B was significantly higher than that in group A. There was a statistically significant difference in the composition ratio of BMI between the two groups (*P* = 0.001) (Table 3).

**Table 3:** Comparison of baseline data in the two periods

Variables	Group A	Group B	*P* Value
Age (years, mean ± SD)	65.4 ± 7.9	65.9 ± 7.4	< 0.001
Sex			0.001
Male, *n* (%)	1641 (21.6)	3759 (23.5)	
Female, *n* (%)	5965 (78.4)	12,245 (76.5)	
BMI (kg/m^2^, *n*, %)			< 0.001
≤ 18.5	91 (1.2)	154 (1.0)	
18.5–23.9	2122 (27.9)	3,673 (23.0)	
24–27.9	3066 (40.3)	6870 (42.9)	
≥ 28	2327 (30.6)	5307 (33.1)	
Kellgren–Lawrence classification			0.02
Grade III	437 (5.7)	1045 (6.5)	
Grade IV	7169 (94.3)	14,959 (93.5)	
Comorbidities			< 0.001
0, *n* (%)	2540 (33.4)	4667 (29.2)	
1, *n* (%)	2787 (36.7)	5398 (33.7)	
2, *n* (%)	1409 (18.5)	3177 (19.8)	
≥ 3, *n* (%)	870 (11.4)	2762 (17.3)	
Surgical strategies			< 0.001
TKA, *n* (%)	7247 (95.3)	15,042(94.0)	
UKA, *n* (%)	359 (4.7)	962 (6.0)	
Hospital stay (days)			< 0.001
TKA (median, interquartile range)	15 (9)	10 (6)	
UKA (median, interquartile range)	10 (6)	9 (6)	
Hospitalization costs ($)			< 0.001
TKA (median, interquartile range)	8441.6 (1338.2)	8346.6 (3120.7)	
UKA (median, interquartile range)	7659.2 (1838.6)	7146.6 (2870.5)	

*SD* standard deviation, *BMI* body mass index, *TKA* total knee arthroplasty, *UKA* unicompartmental knee arthroplasty

### Comparison of changes in K–L classification between the two periods

Regarding K–L classification of the enrolled patients, the number of patients with grade IV in group A was the highest, accounting for 94.3% (7169/7606), followed by patients with grade III accounting for 5.7% (437/7606). In group B, patients with grade IV accounted for 93.5% (14,959/16,004), followed by patients with grade III, accounting for 6.5% (1045/16,004). The proportion of patients with grade III in group B was significantly higher than that in group A. There was a statistically significant difference in the composition ratio of K–L classification between the two groups (*P* = 0.02) (Table 3).

### Comparison of comorbidities between the two periods

Common comorbidities in patients who underwent KA include hypertension, coronary heart disease, diabetes, and cerebrovascular disease. In group A, 66.6% (5066/7606) of patients had comorbidities, among whom 2787 (36.7%) had one medical system comorbidity, 1409 (18.5%) had two, and 870 (11.4%) had ≥ 3. In group B, 70.8% (11,337/16,004) of patients had comorbidities, among whom 5398 (33.7%) had one medical system comorbidity, 3177 (19.8%) had 2, and 2762 (17.3%) had ≥ 3. Compared with group A, patients in group B had more comorbidities, and the difference was statistically significant (*P* < 0.001) (Table 3).

### Comparison of hospitalization-related characteristics between the two periods

In group A, 7247 (95.3%) patients underwent TKA and 359 (4.7%) underwent UKA. In group B, 15,042 (94.0%) patients underwent TKA and 962 (6.0%) underwent UKA. There was a statistically significant difference in the constituent ratio of the surgical procedures between the two groups (*P* < 0.001) (Table 3).

In group A, the length of hospital stay of patients who underwent TKA ranged from 2 to 100 days, with a median of 15 days and an interquartile range of 9 days. The length of hospital stay for patients undergoing UKA ranged from 4 to 34 days, with a median of 10 days and an interquartile range of 6 days. In group B, the length of hospital stay of patients who underwent TKA ranged from 1 to 104 days, with a median of 10 days and an interquartile range of 6 days. The length of hospital stay for patients undergoing UKA treatment ranged from 4 to 38 days, with a median of 9 days and interquartile range of 6 days. There were significant differences in the length of hospital stay between the two periods (*P* < 0.001) (Table 3).

In group A, hospitalization costs for patients who underwent TKA ranged from $1053.70 to $32,426.70, with a median of $8441.60 and an interquartile range of $1338.20. Hospitalization costs for patients who underwent UKA ranged from $1525.20 to $40,048.10, with a median of $7659.20 and an interquartile range of $1838.60. In group B, hospitalization costs for patients who underwent TKA ranged from $1828.30 to $5923.70, with a median of $8346.60 and an interquartile range of $3120.70. The hospitalization costs for patients who underwent UKA ranged from $2393.00 to $20,122.80, with a median of $7146.60 and an interquartile range of $2870.50. There was a statistically significant difference in hospitalization costs between the two periods (*P* < 0.001) (Table 3).

## Discussion

In the present study, we retrospectively analyzed data from 23,610 KOA patients who underwent primary unilateral KA over a 10-year period and found that the number of patients undergoing KA has increased in recent years. Moreover, we compared baseline data, clinical characteristics, and surgical strategies between the two periods to better clarify the changes in recent years. On average, during the entire study period, more than three-quarters of KAs involved females, and the age at surgery tended to be younger than that in foreign countries. The number of patients with overweight, obesity and grade III, and multiple medical diseases increased significantly; however, hospitalization costs and hospital stays have decreased.

To the best of our knowledge, there are many risk factors influencing the occurrence and progression of KOA, including age, sex, obesity, K–L classification, trauma, metabolic dysfunction, environment, and genetic elements [8]. In recent decades, the number of patients with KOA has increased dramatically, and TKA and UKA have been the primary treatment strategies for end-stage KOA [9]. In Finland, Leskinen et al. [10] reported a 130-fold increase in the incidence of TKA among individuals 39–59 years of age between 1980 and 2006. A previous study reported that, from 1991 to 2010, the number of patients who underwent TKA per year in the USA increased from 93,230 to 243,802, corresponding to an increase of 161.5% [11]. According to a recent study, the number of TKAs performed in the USA will reach 1,260,000 annually by 2030. Moreover, the number of TKAs is expected to increase further with increases in arthritis, obesity, and the proportion of elderly individuals in aging societies [12, 13]. Barnes et al. [14] reported that the onset of the COVID-19 pandemic led to a significant reduction in the number of TKAs in the United States Medicare population. In the present study, the number of KAs increased annually during the decade, peaking in 2019, which was 6.18 times higher than that in 2011. The COVID-19 pandemic in 2019 reduced the number of KAs in 2020; it was still five times higher than that in 2011.

Previous studies have analyzed that a number of scholars analyzed the epidemiological characteristics of KA. Chikud et al. [15] conducted a statistical analysis of KOA patients across 926 hospitals in Japan from 2007 to 2010. The results revealed that the average age of patients undergoing TKA was 74.6 ± 6.9 years, with 23.4% of patients > 80 years of age and 68.0% between 65 and 79 years of age. A retrospective study involving patients undergoing TKA in Australia (*n* = 278) reported that the mean age was 68 years (rang, 45–89 years) [16]. Namba et al. [17] conducted an epidemiological study investigating patients undergoing KA surgery in 45 medical institutions across six regions of the USA from 2001 to 2009. Of 56,216 KA patients, of 63.0% of were female, and the mean age was 67.4 ± 9.6 years. Tambascia et al. [18] analyzed data from 122 patients who underwent KA surgery at the Wilson Mero Institute in Sao Paulo, Brazil, from 2009 to 2011. The results revealed that 89 (73.0%) patients were female and 33 (27%) were male, with a mean age of 68.2 ± 8.3 years. In our study, we also found that females had a high incidence of KA surgery (77.1%), with the highest among those 60–69 years of age. The reasons for the higher incidence of KA in women may be related to geographical location, dietary habits, osteoporosis, relatively weak muscle strength, postmenopausal hormone levels, and anatomical structure [19]. A comparison of baseline data from KA patients from 2016 to 2020 and 2011 to 2016 revealed that the proportion of females increased significantly, which is consistent with the of results of domestic and foreign studies. However, the mean age of patients undergoing surgery was 65.7 ± 7.6 years, which tended to be younger than that reported in foreign studies.

Of note, obesity has been a well-established risk factor for KOA [8, 20]. With the acceleration of aging among the population of China, the proportion of obese individuals is also gradually increasing; such, the incidence of KOA will inevitably increase. Namba et al. [17] retrospectively analyzed data from 56,216 patients who underwent primary TKA between 2001 and 2009 at 45 medical facilities across six regions of the USA. The mean BMI was 32 kg/m^2^. Similarly, Belmont et al. [21] searched the NSQIP database and, of 15,321 patients who underwent primary unilateral TKA between 2006 and 2010, 61.2% were obese (BMI ≥ 30 kg/m^2^). In the past 5 years, the proportion of overweight and obesity has significantly increased compared to the previous 5 years, which may be explained by improvements in living standards, caloric intake, exercise reduction, and sedentary lifestyles. At the same time, it has been reported that BMI ≥ 35 kg/m^2^ is a strong risk factor for exercise restriction after KA surgery; moreover, high BMI is closely associated with prolonged hospital stay and readmission rates [22–24]. Therefore, we suggest that high-risk individuals > 60 years of age should consider a reasonable diet, develop good living habits, control their body weight, and engage in adequate levels of exercise. In addition, with the improvement of knee function and quality of life requirements of patients, more and more moderate and severe KOA patients receive surgical treatment [5]. In our study results, it can be shown that the proportion of K–L classification grade III patients receiving KA treatment increased significantly in 2016 to 2020 compared with 2011 to 2015 (6.5% vs. 5.7%, *P* = 0.02).

Presently, there are many related studies investigating the comorbidities of KA patients, mainly including hypertension, diabetes, cardiovascular and cerebrovascular diseases [25, 26]. Chikud et al.[15] performed statistical analysis on inpatients from 926 hospitals in Japan from 2007 to 2010 and found that diabetes was the most common comorbidity among 410,60 patients who underwent TKA, accounting for approximately 16%, followed by chronic lung disease (2.6%) and chronic renal failure (1.9%). In addition, Jones et al. [27] conducted a retrospective analysis of 520 patients who underwent initial KA in a developed region of the USA and found that 13% had diabetes and 24% had heart disease. The results of this study demonstrated that the types of comorbidities were consistent with the results of domestic and foreign studies, and the number of comorbidities showed an increasing trend. However, the proportion of patients with hypertension was highest in China, which is inconsistent with other reports.

Results of our study demonstrated that the median length of hospitalization for TKA and UKA patients was 11 and 9 days, respectively, and hospitalization days were significantly shorter than those in the recent period and the previous 5 years. The median hospitalization cost for TKA patients was $8393.90 and $7319.40 for UKA patients, and costs decreased between the most recent 5 years and the previous 5 years. Many investigators have performed large-sample studies investigating the length of hospital stay and hospitalization costs of KA patients. Bolognesi et al. [28] analyzed 68,603 patients with unilateral KA (65,505 TKA and 3098 UKA) in the USA between 2000 and 2009. The mean length of hospital stay was 3.9 ± 2.1 days for TKA patients and 2.4 ± 1.7 days for UKA patients. Based on an analysis of the National Bureau of Statistics 2009 Inpatient Sample (NIS) database by Pugely et al. [29], the average hospitalization cost and length of hospital stay for TKA were $14,491 and 3.3 days, respectively. Larsen et al [30] studied patients who underwent KA surgery for the first time at Holstebro Regional Hospital in Denmark between August 2005 and February 2007 and found that the hospitalization cost of patients undergoing TKA or UKA was $13,196.60 ± 8481.50. In our study, hospitalization costs were significantly lower than that in other developed countries, however, the total length of hospital stay was significantly longer than that in other developed countries.

It has been established that shortening the length of hospital stay is very important in reducing the economic burden and medical expenses of KA patients [31]. A possible reason for this is that, in recent years, major hospitals in this country have vigorously improved the hospital bed turnover rate, especially by shortening the preoperative wait time, which can directly reduce the number of hospitalization days and expenses. In addition, the preoperative assessment of patient organ function status, management of intraoperative anesthesia and analgesia, and postoperative nutritional support and prevention of complications are also closely related to the reduction in length of hospital stay and costs.

The present study had several limitations, the first of which was its retrospective design, and inherent biases, which may have been unavoidable. Furthermore, because the patients who underwent KA treatment in the two groups were treated in different periods, changes in the medical environment and advances in surgical techniques may have affected effected clinical outcomes. Finally, we only conducted an epidemiological analysis of limited clinical data, some important data such as PSI and robotics alignment were not included. In future studies, more valuable variables need to be collected and analyzed.

## Conclusion

In summary, the changing trends of the epidemiological characteristics of patients who underwent primary unilateral KA were more than three-quarters of KA occurred among females, and the age at surgery tended to be younger than that in foreign countries. The proportion of overweight and patients undergoing UKA surgery has increased. Moreover, compared with 2011 to 2016, the number of patients undergoing KA treatment from 2016 to 2020 complicated with medical diseases increased significantly; however, hospitalization costs and hospital stays showed a downward trend. This study has shed light on the epidemiological characteristics of KA patients in eastern, northern, and central China between 2011 and 2020 and may prove helpful for the prevention of KOA and/or control of its progression.

## Data Availability

The datasets generated during the current study are public at the email 616287291@qq.com.

## Abbreviations

KA: Knee arthroplasty
KOA: Knee osteoarthritis
BMI: Body mass index
TKA: Total knee arthroplasty
UKA: Unicompartmental knee arthroplasty
SD: Standard deviation
COVID-19: Coronavirus disease 2019
